# Shaken Baby Syndrome: Magnetic Resonance Imaging Features in Abusive Head Trauma

**DOI:** 10.3390/brainsci11020179

**Published:** 2021-02-01

**Authors:** Gaia Cartocci, Vittorio Fineschi, Martina Padovano, Matteo Scopetti, Maria Camilla Rossi-Espagnet, Costanza Giannì

**Affiliations:** 1 Emergency Radiology Unit, Department of Radiological, Oncological and Pathological Sciences, Umberto I University Hospital, Sapienza University of Rome, 00198 Rome, Italy; gaia.cartocci@uniroma1.it; 2 Department of Anatomical, Histological, Forensic and Orthopedic Sciences, Sapienza University of Rome, 00198 Rome, Italy; martina.padovano@uniroma1.it (M.P.); matteo.scopetti@uniroma1.it (M.S.); 3 Neuroradiology Unit, NESMOS Department, Sapienza University, 00185 Rome, Italy; mcamilla.rossi@opbg.net; 4 Neuroradiology Unit, Imaging Department, Bambino Gesù Children’s Hospital, IRCCS, 00165 Rome, Italy; 5 Department of Human Neurosciences, Sapienza University of Rome, 00198 Rome, Italy; costanza.gianni@uniroma1.it

**Keywords:** abusive head trauma, neuroimaging, MRI, child, infant, shaking mechanism

## Abstract

In the context of child abuse spectrum, abusive head trauma (AHT) represents the leading cause of fatal head injuries in children less than 2 years of age. Immature brain is characterized by high water content, partially myelinated neurons, and prominent subarachnoid space, thus being susceptible of devastating damage as consequence of acceleration–deceleration and rotational forces developed by violent shaking mechanism. Diagnosis of AHT is not straightforward and represents a medical, forensic, and social challenge, based on a multidisciplinary approach. Beside a detailed anamnesis, neuroimaging is essential to identify signs suggestive of AHT, often in absence of external detectable lesions. Magnetic resonance imaging (MRI) represents the radiation-free modality of choice to investigate the most typical findings in AHT, such as subdural hematoma, retinal hemorrhage, and hypoxic-ischemic damage and it also allows to detect more subtle signs as parenchymal lacerations, cranio-cervical junction, and spinal injuries. This paper is intended to review the main MRI findings of AHT in the central nervous system of infants, with a specific focus on both hemorrhagic and non-hemorrhagic injuries caused by the pathological mechanisms of shaking. Furthermore, this review provides a brief overview about the most appropriate and feasible MRI protocol to help neuroradiologists identifying AHT in clinical practice.

## 1. Introduction

The leading cause of fatal head injuries in children less than 2 years of age is abusive head trauma (AHT), included in the child abuse spectrum, and responsible for roughly 50% of the serious or fatal traumatic brain injury cases [1]. AHT mostly affects infants, male gender, aged between 4 and 6 months, with the peak incidence of fatal AHT between 1 and 2 months of age [2]. The public attention to this type of abuse gradually increased because it represents a devastating illness as well as a problem of social, legal, and forensic importance [3].

The first description of children with chronic subdural hematoma and fractures of long bones with or without retinal hemorrhages dates back in 1946 [4]; the same author later suggested that “whiplash shaking and jerking” are common causes of the skeletal and the cerebrovascular lesion in abused infants [5,6]. Specifically, he showed the effects of rotational acceleration/deceleration of whiplash as the etiology of subdural hematomas and the retinal hemorrhages associated to no/poor external marks of injury. Moreover, Caffey stated that whiplash/shaking may cause repeated and protracted episodes of breath holding with consequent brain damage. The term shaken baby/shaken impact emerged with Duhaime in 1987, who stated that in children with severe injuries blunt trauma must be involved [7]; however, this theory was later questioned by the evidence that shaking alone can be the causative mechanism for severe brain injury [8].

In most recent years both “abusive head trauma” (AHT) and “shaken baby syndrome” (SBS) became accepted terms for diagnosis of non-accidental brain injury in children [9]; however, it has been established that shaking and/or shaking with impact or blunt impact alone can be cause for AHT.

Since the term “shaking” alone was not inclusive of the full range of injury mechanisms, the medical use of the term AHT has been recommended in 2009 by the Committee on Child Abuse and Neglect of the American Academy of Pediatrics as the most comprehensive term for intracranial and spinal lesions [10], and it has been more recently confirmed in a consensus statement from a panel of international experts [11]. Nevertheless, the mechanism of shaking is recognized as the leading mechanism of injury in AHT, with repeated forces in a whiplash-like manner applied on the infant’s chest or extremities causing shaking movements and subjecting the head to continuous accelerations and decelerations, as well as rotational forces.

The highest incidence in infants aged between 2 and 4 months can be justified by several factors: infants mostly living alone with their caregivers, who often fail to cope with the continuous requests and needs of the baby [12]; infants are not able to support the head during shaking movements since the cervical musculature is still developing and strengthening at this age and they have a different body proportion compared to adults, with larger head and heavier brain [13]. Finally, infants have peculiar central nervous system (CNS) characteristics, with immature brain with high water content, neurons only partially myelinated, and prominent subarachnoid space. The characteristic clinical triad of AHT consists of subdural hematoma (SDH), retinal hemorrhage (RH), and hypoxic-ischemic encephalopathy, often in absence of further lesions detectable and without further possible explanations [14].

In about half of the children affected by AHT clinical presentation is acute, and it is characterized by sleepiness, intracranial hypertension, seizures, apneas, reduced muscle tone, anemia, and shock. A hyperacute presentation of brain damage caused by excessive head flexion-extension movements resulting in a brainstem injury can occur; about 6% of abused children present acute respiratory failure and cerebral edema at the time of access to the hospital, resulting almost always in death [15]. Other common features, although less specific for AHT, are long bone fractures often consolidated, newly developed serial paravertebral rib fractures and humerus metaphyseal fractures, both produced during the shaking movement [16], poor appetite, vomiting, fever, irritability, altered state of consciousness, bulging fontanel, apneas, and seizures [17].

Incidence of AHT is established as 14–40 per 100,000 children under the age of 1 year [15]. Diagnosis is not straightforward, and it is based on a multidisciplinary approach with the involvement of several expert professionals in recognizing this kind of abuse [18]. Obviously, antemortem diagnosis is essential to provide the right treatment to the abused infant and to prevent further episodes; similarly, postmortem diagnosis occupies a prominent place, given the mortality rate of AHT ranging from 15% to 38% [1], with the aim of condemning the perpetrator and preventing him from causing the same injuries to other potential victims. Of the surviving infants only 36% of them have good outcomes with the remaining 64% having immediate or future disabilities, from moderate to severe degrees [19]. Indeed, despite the heterogeneity of the studies in terms of time of follow-up, sample size and investigation methods, the long-term impact of AHT has been extensively demonstrated. Specifically, it can be characterized by progressive neurological and cognitive issues; long-term neurological conditions include microcephaly, central hypotonia, spastic hemiplegia, ataxia, dystonia, hydrocephalus, post-traumatic epilepsy, sensorineural deafness, and visual impairment. Long-term cognitive impairment comprises delayed language development, intellectual disability, memory and concentration deficit, poor executive functioning and social skills deficit [20].

As previously highlighted, correct diagnosis represents a medical, forensic, and social challenge. An essential aspect is the acquisition of the anamnesis, with particular attention to the history of the event and the appearance of symptoms; in fact, caregivers often try to justify injuries from falls from heights. Only a few abusers confess that they mistreated the infant in a situation of high stress, for example during an incessant crying of the baby [17]. The ophthalmologic examination and fundoscopy with pupil dilatation are fundamental to study the retina and investigate the presence of hemorrhages. Another test performed is the total body X-ray, with the aim of highlighting new or consolidated fractures. But head computed tomography (CT) and brain and spine magnetic resonance imaging (MRI) are necessary to investigate CNS involvement in infants with suspected AHT [21]; CT and MRI are complementary for diagnosis of AHT. In the past decade efforts have been made to limit the radiation dose administered to children who are more vulnerable than adults to radiation-associated cancer development [22]. “Justification” and “Optimization” principles of ALARA (as low as reasonably achievable) can provide the elimination or reduction of unnecessary/additional radiation exposure associated with CT imaging [23].

CT has a high sensitivity in investigating bone tissue, therefore it is decisive in identifying fracture rhymes (complex skull fractures are common following AHT), but it also detects ischemic areas, areas of cerebral edema, as well as the presence of blood collections. MRI is more sensitive than CT scan in the study of brain parenchyma and it is indicated in cases of suspected AHT with negative CT [24].

The recent consensus statement identified an initial unenhanced CT with 3-D reformatted images of the calvarium [11], followed by a full multisequence MRI of the brain and spine as soon as feasible, as optimal imaging strategy for an acutely ill child with neurologic impairment; on the other hand, children who are intact neurologically can be first imaged using MRI [24,25]. Repeated MRI is often recommended, since timing parenchymal and extra-axial injury can be challenging.

This paper is intended to review the main MRI findings in the central nervous system in infants and young children with AHT, focusing on shaking mechanism that causes both hemorrhagic and non-hemorrhagic injuries. We also provide a brief overview about the appropriate MRI protocol to facilitate the diagnosis of SBS in clinical practice.

## 2. Imaging Features

### 2.1. Hemorrhages

Identifying AHT is challenging because in most cases there are no externally visible injuries and children present nonspecific symptoms. Violent shaking mechanism of the infant might lead to hemorrhagic phenomena, exposing its head to acceleration–deceleration and rotational forces and resulting in retinal and/or different patterns of extra-axial bleeding.

**Figure 1:**
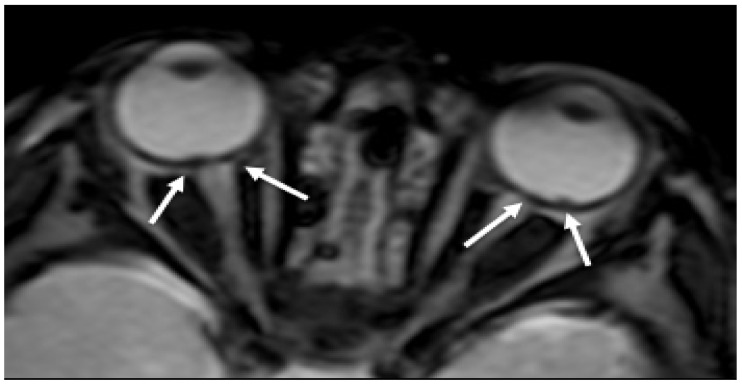
Axial GRE imaging shows irregular thickening and hypointensity of bilateral posterior ocular globes, indicative of retinal hemorrhage (arrows).

RH is a well-recognized manifestation of child abuse found in many babies with AHT (Figure 1). The presence of RH is considered pathognomonic for SBS and is generally associated with more severe neurological damage and a worse clinical outcome [16]. RH is normally detected by fundoscopy. As neuroimaging is always performed to evaluate the central nervous system, RH could be detected as tiny foci of signal dropout of ocular globes along the retina on gradient echo T2 (GRE T2-w) or susceptibility weighted imaging (SWI) sequences and need to be carefully looked for [26]. Although the sensitive detection of blood involves susceptibility imaging sequence, specificity for timing and aging of the hemorrhage is low.

**Figure 2:**
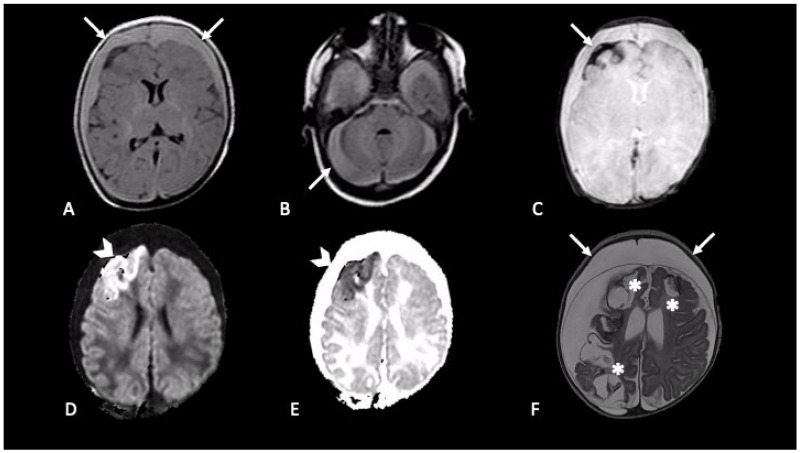
Newborn first accessed in the emergency department with fever and vomit, then was hospitalized four days later due to generalized seizures. CT showed mixed-density bilateral fluid collections (not shown). MRI demonstrated bilateral subdural CSF-blood collection, supra- and infra-tentorial (**A**,**B** axial FLAIR, arrows); subarachnoid blood products are revealed by susceptibility weighted imaging in right frontal lobe (**C**, axial SWI, arrow); intraparenchymal cortical-subcortical hemorrhage in the right frontal lobe (**D**,**E**, axial DWI and ADC map, arrows-head). One-month follow-up MRI (**F**, axial T2) showed malacic evolution of intraparenchymal damage (asterisks) and bilateral subdural hygromas (arrows).

**Figure 3:**
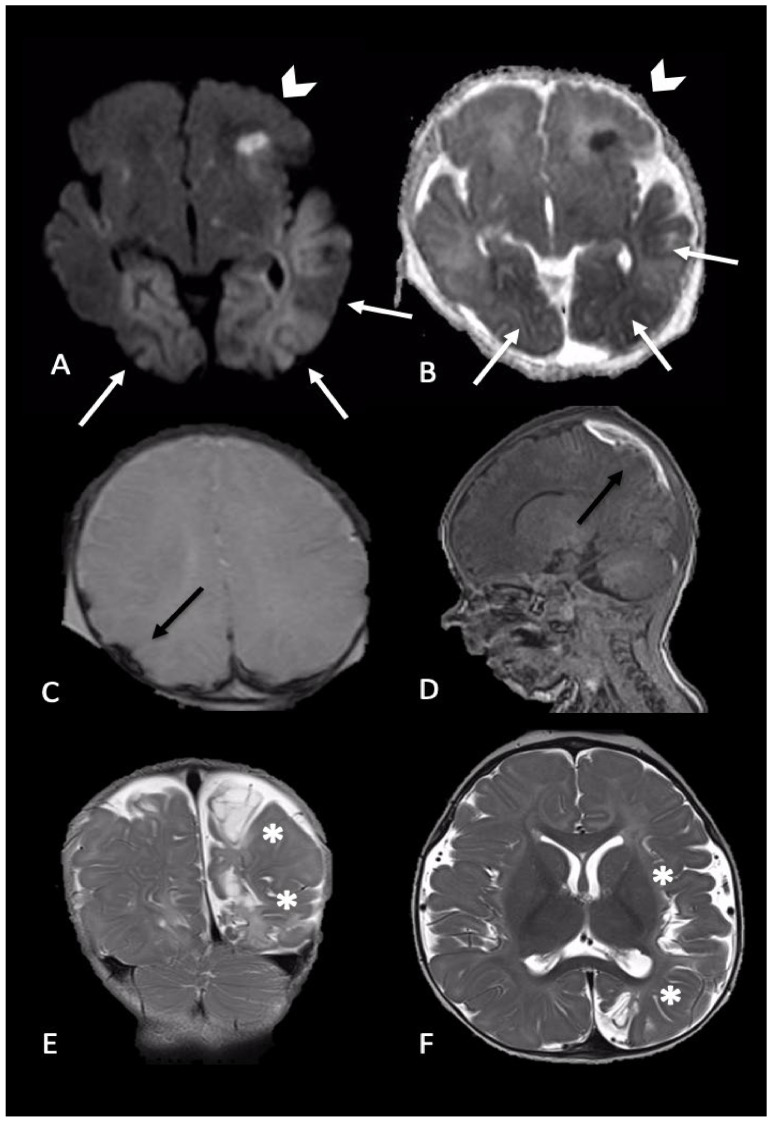
Newborn reached the emergency department for single episode of apnea followed by generalized seizures. MRI performed 4 days later showed intraparenchymal injuries and bilateral extra-axial mixed-intensity fluid collections. In particular, (**A**,**B**) (axial DWI and ADC maps) showed bilateral temporal and occipital cortical areas of restricted diffusion (white arrows) and a focal area of restricted diffusion localized in the left frontal lobe and diffuse (arrows-head), indicative of acute intraparenchymal injury; (**C**,**D**) (axial SWI and sagittal T1-W) showed extra-axial blood collection along the parietal convexity bilaterally (black arrows). Six-month follow-up MRI ((**E**,**F**), coronal and axial T2-W) revealed malacic evolution of intraparenchymal damage and consequent expansion of extra-axial spaces due to tissue loss (asterisks).

Extra-axial hemorrhages are commonly observed in AHT and can occur in any of the three major anatomic compartments, as epidural, subdural, and subarachnoid space [20]. The neuroradiological finding that usually raises suspicion of SBS, especially when found in association with RH and inappropriate clinical history, is SDH [27] (Figure 2 and Figure 3). Although SDH is the most common finding in SBS, the presence of SDH itself does not prove the syndrome. SDH should be carefully evaluated along with clinical history and physical examination to differentiate other possible causes [28]. For example, in the first few months of life, parturitional SDH cannot be differentiated by inflicted trauma, based on imaging findings alone. To discriminate those two mechanisms patient age, birth history, and delivery method (forceps, caesarean etc.,) must be investigated [29].

SDH in AHT has typical localizations over the convexities (unilaterally or bilaterally), in the interhemispheric space or within the posterior fossa and may not be associated with skull fractures or scalp hematomas [30]. The violent shaking of the infant can lead to the tearing of bridging veins, due to acceleration–deceleration and rotational forces on the infant’s head [31]. Bridging veins are located at the vertex and cross the arachnoid space perpendicular to the superior sagittal sinus. Therefore, they are particularly vulnerable to rupture during antero-posterior movements [32]. Signs of ruptured bridging veins have been previously described as a sign of the traumatic cause of SDH in suspicious of AHT [27,33]. GRE T2-w and, more accurately, SWI sequences have been reported to be sensitive for blood products and are thought to be very helpful in the identification of clot formation on injured bridging veins. The predominant (73%) bridging veins thrombosis shape was found to be the “Tadpole Sign”, where the “body” of the tadpole corresponds to the clot derived from the injured bridging veins expanded by clotted blood (considered as a “tail of the tadpole”) [34].

Since the clinical history is often unreliable, information about the timing of the injury must be hypothesized from neuroimaging. Compared to CT, age determination of SDH can be estimated with better accuracy with MRI, especially for small blood collections [35]. After bleeding, blood products rapidly desaturate and fully oxygenated hemoglobin is converted to deoxyhemoglobin and then to methemoglobin, which contains ferric iron. As red blood cells lyse, methemoglobin is released and eventually degraded and resorbed. Macrophages convert the ferric iron into hemosiderin and ferritin [36]. These molecules can be characterized by their magnetic susceptibility effect [37]. Determining the age of subdural blood products can be challenging and does not follow the rules of evolution described in parenchymal hemorrhages at MRI because elevated tissue thromboplastin and oxygen concentration lead to a more rapid breakdown of hemoglobin in the latter [38].

Bradford et al. retrospectively identified different patterns of SDH in a group of 43 infants and correlated them with time interval between injury and MRI scans [39]. In brief, authors of this study assert that homogeneous SDH is represented by early/late subacute MR findings (T1 hyperintensity; T2/FLAIR hypointensity at early stage and T2/FLAIR hyperintensity at late stage), while heterogeneous SDH reflects a mixture of different intensities (Pattern III contains equal mixtures having different intensities, whereas Pattern IV has fluid that is predominantly T1 hypointense and T2/FLAIR hyperintense) [39]. Mixed intensity presenting within different regions of the collection on the same MR image can represent the inhomogeneous distribution of fluid of different composition within the hematoma cavity (blood products and CSF) or can be the result of blood products of different ages.

Re-bleeding of chronic SDH must be suspected in presence of focal high signal on T1-w images. This phenomenon is due to friable new capillary beds characterizing chronic SDH, that can predispose to such rebleeds [40]. Acute-on-chronic subdural fluid collections of varied signal intensity separated by internal membranes might represent hemorrhage caused by multiple traumatic episodes or in case of repeated shaking injury without trauma, as the brain is pulled away from the cranium and the bridging veins are stretched and torn [41]. Gadolinium administration can assist in distinguishing acute-on-chronic hematomas by helping to identify membranes within the subdural fluid. The presence of internal membranes is an important key point in order to differentiate chronic SDH from subdural hygromas, which is a fluid collection of predominantly CSF [42].

Subdural hygromas represent the final stage of SDH, as deposited blood products remnants and it could be interpreted as a later consequence of AHT, which occurred a few weeks before [43]. Zouros et al. proposed a mechanism whereby subdural hygromas directly originate from shaking the baby: shear forces caused by acceleration/deceleration of the brain may disrupt bridging veins and Pacchioni’s granulation, resulting in a free CSF communication between subarachnoid and subdural space [44]. In conclusion, chronic SDH and subdural hygromas are often difficult to distinguish from each other and could be wrongly used as synonymous in daily clinical practice. Contrary to adults, chronic SDH in infants are infrequent compared to subdural hygromas and radiologist should gain experience and ability from principal MR findings to distinguish different causes of subdural collection in suspected AHT.

Subarachnoid hemorrhage (SAH) represents another common, though non-specific, neuroradiological finding that could be detected in AHT [28] (Figure 2). In shaking injury, tearing of small vessels in the pia and arachnoid causes SAH, more often seen along the high cerebral convexities or within the interhemispheric fissure. In this location it is really challenging to radiologically distinguish SAH from SDH, and those two entities (SDH and SAH) may coexist here [21]. “Blooming artifact” on susceptibility imaging sequences (SWI or GRE T2-w), and FLAIR are principally used to detect SAH.

Compared to SDH and SAH, epidural hematoma (EDH) is uncommon in SBS. In EDH blood accumulates beneath the inner skull table and above the dura after a blunt impact trauma and consequent rupture of the middle meningeal arteries [37]. Skull fracture can be present and the trauma history should be carefully evaluated in these suspected cases [28].

### 2.2. Parenchymal Injury

**Figure 4:**
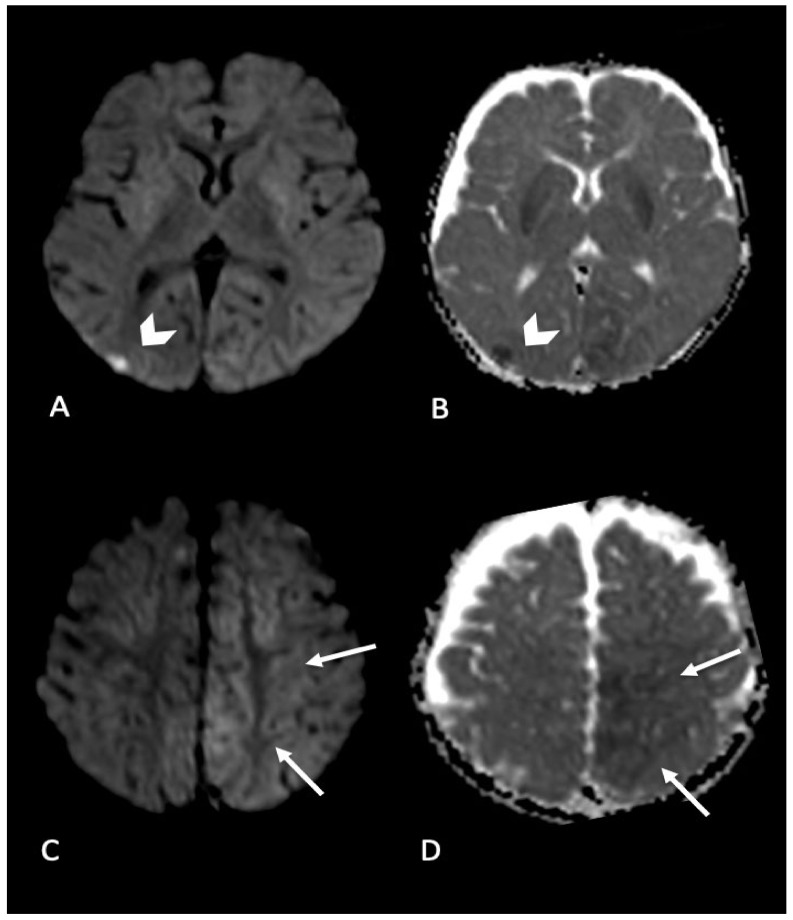
Infant found with right side focal seizures with secondary generalization. Bruises on the right ear, retinal hemorrhages and subdural hemorrhages. MRI performed in the emergency department showed bilateral parenchymal injury. In particular, in (**A**,**B**) (axial DWI and ADC map) diffusion weighted imaging revealed focal area of restricted diffusion in right occipital cortex (arrow’s head); (**C**,**D**) (axial DWI and ADC map) showed areas of restricted diffusion in left mesial parietal cortex (arrows).

Parenchymal brain injury is the most significant cause of morbidity and mortality in AHT; it may be direct mechanical such as contusion, direct axonal injury, laceration, or parenchymal hematoma or indirect due to hypoxia and ischemia. It can be also categorized as focal or diffuse, unilateral or bilateral (Figure 2, Figure 3 and Figure 4). MRI is more sensitive than CT in delineation of parenchymal injuries.

Diffuse parenchymal injury, although not specific for abusive head trauma and rarely reported as isolated finding, is however highly associated with other MRI findings in the abusive head trauma [19,30]. Since its presence has been demonstrated only in trauma caused by high-force mechanisms, thus mostly excluding accidental causes [45], diffuse parenchymal injury is highly suggestive for AHT. Moreover, it is the imaging finding most predictive of the clinical and neurodevelopmental outcome [46,47,48].

Diffuse brain swelling and vascular congestion leading to neuronal death are common findings in children with different grades of encephalopathy due to AHT ranging from irritability to coma or death [49,50]. Both vasogenic and cytotoxic edema are responsible for diffuse brain damage in AHT. Edema can cause obstruction of cerebral artery which leads to perfusion failure and ischemia [49]. However, the pathophysiology of the diffuse parenchymal injury in the shearing AHT has not been completely elucidated yet. Traumatic diffuse axonal injury (DAI) resulting from shear forces due to shaking has been considered the predominant parenchymal injury in children with AHT [51,52] but most recent studies have reported the hypoxic-ischemic injury (HII) as the most frequent cause of parenchymal injury, suggesting unmyelinated axons in the immature brain as a mechanism of resistance to traumatic axonal damage [50,53,54].

In fact, HII is very frequently reported in children with AHT (up to 97% of cases) [55] and seems to be multifactorial; it may result both from direct traumatic injury with oxidative stress and excitotoxicity probably due to seizure activity [56] and from focal traumatic axonal damage at the cervico-medullary junction with consequent apnea [57,58]. However, cervico-medullary injury with apnea does not explain the unilateral HII, often described in AHT [46,59]. Two interesting hypotheses have been made as possible pathophysiology mechanisms of unilateral HII; transient unilateral vascular occlusion [57] and the “second impact syndrome” characterized by unilateral subdural hemorrhage (SDH) and ipsilateral HII, originally reported in young adults with repetitive head trauma related to sports [60]. Second impact syndrome has been related to altered cerebral autoregulation, possibly directly ascribed to the presence of SDH.

Diffuse bilateral parenchymal brain edema due to shaking, and parenchymal damage in different vascular territories are frequent in AHT and are related to a poor prognosis [61]. Diffuse swelling and consequent loss of cerebral blood flow autoregulation also sustain further hypoxic damage [62]. Pathophysiological mechanisms underlying stroke remain however unclear; strangulation and direct stretching of the neck have been suggested as possible causes of infarction in the territory of distribution of the carotid artery and arterial dissection, respectively [63]. Fat embolism in the context of long-bone fractures and direct damage to arterial wall due to shaking mechanism can also cause stroke [61]. Cystic areas and gliotic scars can occur in the sites of initial edema at follow-up MRI examinations; the more rapid the development of multicystic degeneration and or gliotic scars, the more severe is the neurodevelopmental outcome after hypoxic-ischemic injury [48,61]. However, gliotic scars found 9–12 months later the initial injury are usually smaller and not related to a poor neurodevelopmental outcome.

MRI is more sensitive than CT in detecting early diffuse parenchymal injury related to AHT. Among the available sequences, diffusion weighted imaging (DWI) is more effective than T2-weighted images to demonstrate the amount of cytotoxic edema in the unmyelinated brain of infants [64]. DWI is highly sensitive in the identification of foci of acute shear injury, so it is the sequence of choice for the early detection of cytotoxic edema in DAI and HII, allowing the differentiation between reversible vasogenic (high ADC values) and irreversible cytotoxic (low ADC values) edema [65,66] (Figure 4). Moreover, the volume of DWI signal abnormalities correlates better than any other imaging variables with both the acute Glasgow Coma Scale Score and a subacute outcome ranking scale score [67]. Several authors evaluated that the incidence of HII in AHT with DWI ranges from 30% to 40% [57,68,69]. DWI may depict brain areas of restricted diffusion in AHT, compatible with arterial stroke, with global hypoxic event as the major responsible. In a recent study, infants among 1 and 6 months of age showed the highest incidence of stroke, mainly bilateral, multifocal, and often associated with SDH [70]. In the same series of patients, some children presented with venous stroke, following cortical vein thrombosis.

DWI helps also in distinguishing between DAI and vascular injury based on the territory of distribution of the low ADC areas. A watershed pattern of restricted diffusion has been often reported in AHT [57,64,68]. The more severe form is bilateral total supratentorial diffuse cortical and subcortical swelling [68], probably representing a more severe form of the watershed pattern of HII. Recently, Orru and colleagues described two distinct DWI patterns of HII, (i) asymmetric bilateral cortico-subcortical (ii) diffuse cortical and deep GM, predictive of worse clinical outcome, even if not correlated with SDH or fractures [69]. Finally, an important aspect to be taken into account when dating the parenchymal injury related to AHT is that the timing of the evolution of DWI signal may be delayed in infants compared to ischemic injury in adults. This difference is related to the specific pathologic substrate HII in the developing brain such as autophagy, delayed apoptosis, acute necrosis, and necrosis-like cell death [71].

Diffusion tensor imaging (DTI), an advanced technique derived from the conventional DWI that allows the identification of the directionality of the water molecules movement, revealed widespread microstructural alterations in the white matter i.e., a reduction in axial and mean diffusivity [72] probably reflecting hypoxic-ischemic axonal injury, with a higher prognostic value for clinical outcome compared to conventional DWI.

Focal parenchymal injury in AHT is less common and less characterized than diffuse parenchymal injury and can be due to both shearing injury and direct impact. If caused by shearing mechanisms, focal parenchymal injury varies according to the age of patients, mainly because of different stages of myelination process. Indeed, in children younger than one year of age parenchymal lacerations or contusional tears have been described as characteristic of AHT [73]. Multifocal parenchymal lesions characteristic of traumatic DAI is uncommon in older children [68].

Even if not specific for AHT, DAI has been reported as more frequent in AHT compared to accidental brain injury [74]. DAI is classically defined as axonal swelling due to sudden acceleration/deceleration forces [75]. Multiple micro-hemorrhages at the interface GM/WM and along the WM tracts, as corpus callosum and cerebral peduncles [41] are the key imaging findings in DAI. T2*- GRE and SWI are the sequences of choice for detecting micro-hemorrhages, as hypointense foci with/without adjacent edema [76]. Colbert and colleagues correlated the presence of intraparenchymal micro-hemorrhages on SWI sequence with poor long-term neurological outcomes [77]. DAI is also common at the level of the respiratory centers of the medulla, resulting from forces exerted on these structures during violent shaking of the head, when the neck muscles offer little physical support. On the other hand, DAI is not frequent in the supratentorial brain [53,68].

Parenchymal lacerations, also known as subcortical cleft, contusional clefts or tears and gliding contusions, are not common in infants but have been reported in AHT [78,79]. Radiologist should always carefully inspect the subcortical WM especially in frontal lobes, looking for linear intraparenchymal cavities/disruption or clefts containing cerebrospinal fluid and/or blood products; surrounding edema can be present [75,78]. Some authors reported these lesions as pathognomonic of shaking injury [80]. More recently, high incidence of parenchymal lacerations has been reported in AHT compared to accidental injury [79]. They can be also associated to subpial hemorrhage. FLAIR and SWI imaging are more sensitive for parenchymal lacerations detection, the former especially in children older than two years of age, when majority of myelination is complete [81]. Finally, a multifocal punctate diffusion restriction pattern has been rarely descripted in AHT [68].

### 2.3. Spinal Injury

The presence of spinal injury in AHT is an underestimated condition. So far, only a few studied have reported spinal injury in children exposed to AHD with an extremely variable incidence ranging from 15% to 78% of cases with different type of injuries: ligamentous, spinal cord, subdural spinal hematoma, or bone injury. This variability is probably due to several factors including the different period of time retrospectively analyzed, the timing of MRI scan, the differences in image protocols with variable spinal tracts included in the scanner, and different types of spinal injury investigated.

Several anatomic factors predispose the neonatal and infantile spine to different types of injury in case of trauma such as shaking. Among the predisposing factors the most important to consider are the high head/neck size ratio, the poor muscle tone and head control, the presence of horizontally oriented facet joints with poorly developed and cartilaginous joints, the short spinous and transverse processes, and the unique curvature of the spine with fulcrum at the C2-C3 level. All these factors make the neonatal and infantile spine more vulnerable to external forces such as hyper-flexion and hyper-extension and increase the risk for spinal cord injury especially at the level of the cranio-cervical junction and cervical spine.

**Figure 5:**
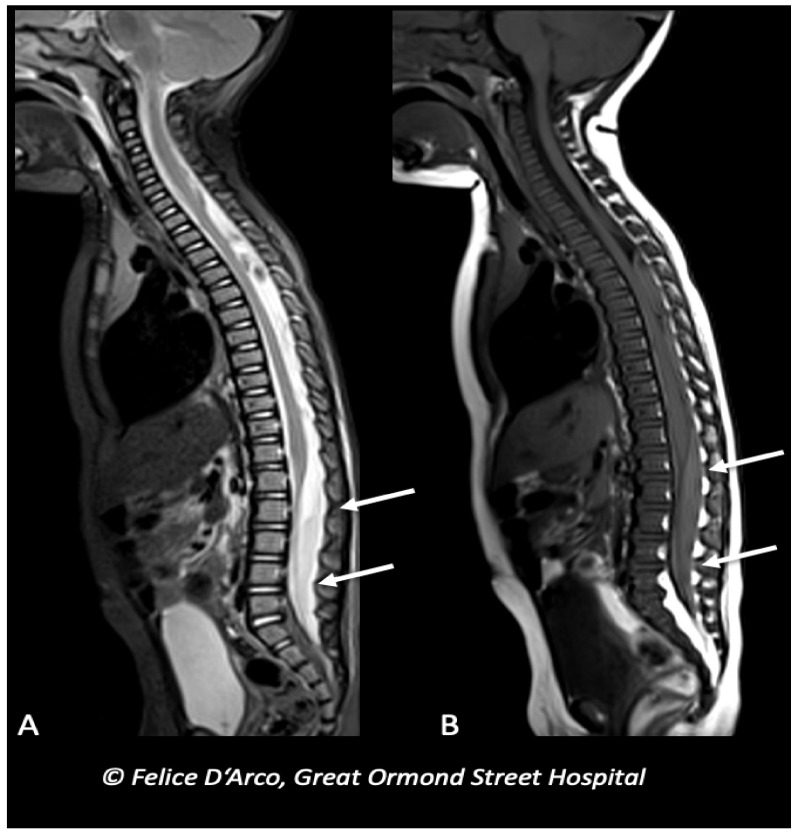
Infant with multiple long bones and ribs fractures (X-ray not shown), and bilateral subdural blood collections at the convexity (brain MRI not shown), subsequently underwent MRI spine examination showing extra-axial mixed-intensity fluid collection indicative of subdural hemorrhages at the level of lumbar spine (arrows) (**A** and **B**, sagittal T1 and T2).

One of the most frequent spinal findings in AHT is the presence of spinal SDH (Figure 5). Several theories have been postulated to explain the presence of spinal SDH in absence of other spinal lesions. Some authors have hypothesized that it may be due to the transmission of the kinetic energy produced by repeated shaking from the posterior ligamentous injury to the underlying spinal canal via myo-dural bridges, with consequent enlargement of the spinal subdural space. This will in turn favor the migration of SDH from the posterior cranial fossa to the spinal canal [82]. On the other hand spinal SDH may be the consequence of direct injury to vessel within the intradural compartment [83,84].

However, it is difficult to speculate about the physio-pathologic process leading to spinal SDH as MRI of the spine is frequently acquired with a different timing compared to the brain and often only the cervical spine is included in the study.

Choudhary et al. found a significant higher difference in the incidence of spinal SDH in children exposed to AHT compared to accidental trauma [85]. The authors reported an increased incidence in MRIs of AHT where the thoracolumbar spine was included in the protocol (63%) compared to those where only the cervical tract was imaged (24%), thus highlighting the importance of including the entire spine in the standard MRI protocol of AHT [85]. Moreover, in all cases there was an association of spinal SDH with intracranial SDH supporting the theory of possible tracking of intracranial SDH into the spinal compartment by gravity.

Also, cervical ligamentous injury has a higher incidence in children with AHT compared to those with accidental trauma [86]. The majority of lesions involve the nuchal ligament and the interspinous ligament, particularly at the level of the cervical spine [86]. These evidences confirm the importance of including the spine in standard MRI protocol for AHT and to include STIR sequences to favor the detection of ligamentous injuries.

Spinal cord injury has a low prevalence in imaging studies on AHT [87] and has been mainly reported in post-mortem studies at the level of the cranio-cervical junction [88]. The reported cases of spinal cord injury in AHT are often not associated to vertebral fractures but are directly related with the severity of the brain injury [88].

Due to the specific anatomic features of the neonatal and infantile spine, which is characterized by high flexibility and cartilaginous joints, spinal fractures are extremely uncommon in AHT patients [84,89].

## 3. MRI Protocol

Conventional MRI as T1-weighted, T2-weighted, and GRE T2-weighted sequences should be always performed in clinical suspect of SBS. A growing body of evidences have shown the superiority of MRI in identifying central nervous system involvement [15]. Specifically, MR is more sensitive to detect brainstem and cerebellar injury, subtle parenchymal injury, ischemic damage, diffuse axonal injury, and to assess timing of extra-axial hemorrhages [62].

In clinical practice MR protocol should always include an axial and coronal T2-w, a sagittal T1-w and an axial GRE T2-w to discriminate the presence or absence of parenchymal or extra-axial hemorrhage; moreover, the combination of these three sequences better elucidates blood products age. Moreover, T2-w sequences acquired at least in two planes accurately evaluate parenchyma. High resolution T2 3D-w sequences can represent an alternative to obtain multiplanar images in a fast modality [28]. Some authors recommend gadolinium-enhanced imaging to demonstrate subdural membranes or possible leptomeningeal reaction [90].

DWI and the corresponding apparent diffusion coefficient (ADC) may have a fundamental role in evaluating AHT, specifically for the assessment of parenchymal injury [91]. DWI is usually acquired on the axial plane and b = 1000 s/mm^2^ is the minimum required gradient strength. Qualitative evaluation is performed on trace images, while quantitative assessment is based on the parameter ADC (high signal on trace images and low signal on ADC meaning restricted diffusion respectively). DWI is the most sensitive sequence in detecting early parenchymal acute cytotoxic edema, hours before changes on T2-FLAIR images and days before changes on CT [76] and also identifies new areas of brain injury weeks after initial injury. Hypoxic-ischemic injuries are common in AHT and correlate with poor clinical outcome in the long term [57]. Low values in ADC maps also correlate with poor long-term neurodevelopmental outcomes [92]. DWI often identifies more extensive damage compared to conventional T2-FLAIR images [93] showing different pattern of injury: diffuse supratentorial, DAI, venous infarction, arterial watershed hypoxic ischemia, parenchymal contusions [68].

SWI is an MRI technique that is sensitive to blood products such as deoxyhemoglobin, methemoglobin, and hemosiderin as well as calcium and iron depositions. It is used to depict microbleeds, subarachnoid siderosis, and clot formations in the diagnosis of different diseases, and has been shown to be more sensitive than conventional GRE T2-w [94]. Since conventional GRE T2-w has demonstrated low sensitivity compared to CT for intracranial hemorrhages, especially for small subarachnoid hemorrhage [95], more advanced GRE T2-w as SWI can be considered. SWI is particularly sensitive to substances which determine local magnetic field distortion, such as calcium and blood products. Sensitivity of SWI in identifying intracranial hemorrhages was recently found to be equal to CT in children younger than 7 years [96].

MR spectroscopy (MRS) is used as a metabolic assay to describe biochemical changes during the evolution of neuronal injury in infants after SBS. MRS is useful to evaluate the presence of lactate and lipids, which has been correlated with worse prognosis [97].

Entire spine examination, with T1-w, T2-w, and STIR sequences must be performed to detect cervical ligamentous injury or spinal SDH [85,86].

Fast MRI protocol is frequently used in pediatric hospital, specifically in an emergency setting, with the aim of avoiding radiation exposure and anesthesia [98]. While T2-weighted sequences are routinely used for the evaluation of hydrocephalus, fast MRI protocol including other sequences should be validated in the assessment of SBS in suspected AHT. Among other studies, Berger had recently validated a fast protocol which lasts 6 minutes, including axial ssT2FSE, axial GRE T2-w, coronal T1-weighted inversion recovery and DWI [99]. However, interpretation of studies on rapid MRI and evidence-based assessment of sensitivity and pitfalls are not straightforward because of small sample sizes and variability in the technique across different study protocols. Moreover, fully replacing CT with fast MRI decreased the sensitivity in detection of skull fractures and missing additional sequences as venographic imaging for bridging vein rupture and spine imaging for spinal cord injuries [100]. Taken all together, data about rapid MRI are promising for screening when AHT is highly suspected in well-appearing children, but caution is mandatory and further imaging is often required for detecting more subtle findings.

## 4. Conclusions

Since AHT represents a major cause of fatal head injuries in infants and young children, a prompt diagnosis is highly recommended to prevent mortality and long-term disability. Diagnosis is multidisciplinary, it is primarily based on an accurate clinical history, and requires neuroimaging to demonstrate the evidences of brain and spine injury.

Among hemorrhagic features, retinal hemorrhages and SDH are very frequent and highly suggestive of AHT, with bridging vein rupture and thrombosis commonly associated. Different stages of subdural collections and possible re-bleeding of chronic SDH need to be also assessed as they are related to the time interval between the injury and MRI. Although less frequent, SAH, EDH, and subdural hygromas can be present and need to be recognized and reported. Parenchymal injury, either direct mechanical such as contusion, direct axonal injury, laceration, and parenchymal hematoma, or indirect due to hypoxia and ischemia, can be focal or diffuse. Diffuse brain swelling due to either traumatic diffuse axonal injury or hypoxic-ischemic injury is frequent in AHT and related to a very poor outcome. Focal parenchymal injury is less common and can be due to both shearing injury and direct impact. Finally, parenchymal lacerations, also known as subcortical/contusional cleft, or tears can be present. Since the presence of spinal injury in AHT is underestimated, MRI protocol for AHT should always include the entire spine to look for SDH, cervical ligamentous, and spinal cord injury.

Applying the proper imaging protocol and being familiar with both typical and atypical findings of AHT are essential for the radiologist to support the clinician in making a correct diagnosis.

## Data Availability

Data sharing not applicable to this article. No new data were created or analyzed in this study.
